# Developing a Deep Brain Stimulation Neuromodulation Network for Parkinson Disease, Essential Tremor, and Dystonia: Report of a Quality Improvement Project

**DOI:** 10.1371/journal.pone.0164154

**Published:** 2016-10-06

**Authors:** Richard B. Dewey, Padraig E. O’Suilleabhain, Manjit Sanghera, Neepa Patel, Pravin Khemani, Laura H. Lacritz, Shilpa Chitnis, Louis A. Whitworth, Richard B. Dewey

**Affiliations:** 1 Department of Neurology and Neurotherapeutics, University of Texas Southwestern Medical Center, Dallas, Texas, United States of America; 2 Department of Psychiatry, University of Texas Southwestern Medical Center, Dallas, Texas, United States of America; 3 Department of Neurological Surgery, University of Texas Southwestern Medical Center, Dallas, Texas, United States of America; University of Medicine & Dentistry of NJ - New Jersey Medical School, UNITED STATES

## Abstract

**Objective:**

To develop a process to improve patient outcomes from deep brain stimulation (DBS) surgery for Parkinson disease (PD), essential tremor (ET), and dystonia.

**Methods:**

We employed standard quality improvement methodology using the Plan-Do-Study-Act process to improve patient selection, surgical DBS lead implantation, postoperative programming, and ongoing assessment of patient outcomes.

**Results:**

The result of this quality improvement process was the development of a neuromodulation network. The key aspect of this program is rigorous patient assessment of both motor and non-motor outcomes tracked longitudinally using a REDCap database. We describe how this information is used to identify problems and to initiate Plan-Do-Study-Act cycles to address them. Preliminary outcomes data is presented for the cohort of PD and ET patients who have received surgery since the creation of the neuromodulation network.

**Conclusions:**

Careful outcomes tracking is essential to ensure quality in a complex therapeutic endeavor like DBS surgery for movement disorders. The REDCap database system is well suited to store outcomes data for the purpose of ongoing quality assurance monitoring.

## Introduction

Treatment of disorders such as PD, ET, and dystonia requires an individualized, multi-faceted approach consisting of non-pharmacological therapy, medications, and surgical treatments. Currently, high frequency deep brain stimulation (DBS) is the most commonly recommended surgical approach when response to medication is inadequate [1, 2]. DBS consists of an implantable neurostimulation system that creates a non-destructive and reversible disruption of the abnormal activity in the basal ganglia or thalamus to improve motor symptoms [3, 4]. Selection of the target is based on disease specific considerations including the patient’s most disabling symptoms, as well as co-morbid cognitive and mood symptoms. Once the system is implanted the device is programmed to deliver electrical current to the targeted area.

Randomized trials of DBS in PD report a range of outcomes. A recent review tabulated the results of a number of studies of both STN and GPi DBS [5]. Of the 9 studies reporting on 943 patients undergoing STN DBS, the mean improvement in UPDRS III scores and PDQ-39 index scores ranged from 29–49% and 8.3–26.4% respectively. The same outcome measures for GPi DBS performed in 377 patients showed mean improvements of 29–39% and 6.3–17.5%. Possible reasons for a variable response, even at pioneering hospitals where pains have been taken to optimize the processes, include patient differences such as anatomy and physiology at the millimeter scale and operational differences such as the trajectory planning process and the heuristics used to decide a lead location is good enough to quit searching for better.

During the early years of our DBS practice, we followed published guidelines on patient selection, surgical technique, and post-operative programming, but there was no systematic collection and review of the outcomes, so there was a missed opportunity to learn based on performance. In the course of routine clinical care we discovered 2 cases of suboptimal clinical responses to DBS, and post-operative imaging confirmed that the leads were not optimally located. These findings prompted a closer look at our DBS program, leading to a quality improvement initiative to refine patient selection, imaging, surgery, and post-operative programming to achieve more consistently positive outcomes.

We have implemented Plan-Do-Study-Act (PDSA) cycles as recommended by the Agency for Healthcare Research and Quality for continuous improvement of each component of the DBS pathway [6]. Parallel and sequential PDSA cycles were undertaken with goals of improving each part of the DBS program as well as the performance of the overall program. In a PDSA cycle, a goal is chosen and literature is reviewed for how best to accomplish this; a process is selected and implemented; results are studied to ascertain if the goal is being accomplished, and if so, the process is retained and another PDSA cycle initiated in another area, but if not, the planning step is undertaken again, this time selecting another change in process aimed at reaching the goal. Herein we report the initiative we have undertaken in our practice to systematically improve surgical outcomes through the processes of patient selection, implantation of the DBS lead, and post-operative management.

## Methods

In order to implement a series of PDSA cycles, we first needed to develop a formal programmatic structure to enable reproducibility, standardization, and continuous monitoring of outcomes. This new structure we termed the “neuromodulation process” begins with implementation of a checklist of procedures that each patient must undergo before being considered for surgery. As patients complete preoperative testing, the results are compiled in a REDCap^™^ database for review by the neuromodulation committee at monthly meetings. Entry of patient data into this research database was conditioned upon written informed consent of the patient and was approved by the UT Southwestern Institutional Review Board. REDCap (Research Electronic Data Capture) is a secure, web-based application designed to support data capture for research studies, providing 1) an intuitive interface for validated data entry; 2) audit trails for tracking data manipulation and export procedures; 3) automated export procedures for seamless data downloads to common statistical packages; and 4) procedures for importing data from external sources [7]. To evaluate outcomes from PDSA cycles, we query the database to analyze changes in motor and non-motor clinical scores which are recorded longitudinally from baseline to 5 years postoperatively.

The results of our QI project (following the implementation of multiple PDSA cycles in each area) are the process enhancements of the following elements of the DBS practice: 1) patient selection, 2) clinical scoring and database tracking, 3) cognitive testing, 4) brain imaging, 5) neuromodulation network meeting, 6) the surgical procedure itself, 7) post-operative programming, and 8) outcomes review meetings which we describe below. In addition, we present preliminary outcomes data on all patients receiving surgery since the development of our outcomes tracking database.

## Results

### Patient selection

DBS at our institution is standard of care for PD patients with disabling motor fluctuations and dyskinesias despite medication optimization, severe medication-refractory tremor, or intolerance to medication [8, 9]. Patients who meet these criteria are counseled about motor features amenable to DBS as well as those which are typically unresponsive to surgery such that patient expectations are commensurate with established outcomes [10, 11]. Similarly, patients with essential tremor and dystonia are selected who are likely to do well with DBS surgery and who have failed appropriate medication trials [12, 13]. The potential risks of surgery are explained in detail for all diagnoses. Those interested in pursuing DBS are given a checklist of procedures and educational materials specific to the condition being treated (S1 Appendix). Next, the evaluating movement disorders neurologist arranges 1) preoperative neuropsychological testing, 2) preoperative brain MRI scan, 3) appointment with the DBS clinical coordinator for scoring, 4) physical therapy evaluation where the mini-BESTest [14] is performed, and 5) a speech and swallowing assessment where the Consensus Auditory-Perceptual Evaluation of Voice (CAPE-V) [15] and the Voice Handicap Index (VHI-10) [16] are performed followed by a swallow evaluation and laryngeal videostroboscopy. Patients with speech and swallowing impairment are educated about the potential worsening of these deficits after DBS and appropriate treatment is initiated prior to surgery.

### Clinical scoring and database tracking

The DBS coordinator meets with the patient preoperatively and obtains written informed consent from all participants to record their clinical data in the REDCap database. The coordinator then performs the following clinical assessments (for PD patients): 1) Movement Disorder Society revision of the Unified Parkinson Disease Rating Scale (MDS-UPDRS) [17] parts 1–4; part 3 is performed 12 hours off and following a clinically effective dose of levodopa in the on state (both are videotaped for later analysis), 2) Parkinson’s Disease Questionnaire (PDQ-39) [18], and 3) Quick Inventory of Depressive Symptomatology, Self Report (QIDS-SR_16_) [19]. These scales are repeated at all postoperative visits along with a clinical global impression scale rated by the DBS programming neurologist and the patient using a 10 point Likert scale (S2 Appendix). At postoperative visits, MDS-UPDRS part 3 is performed and videotaped in the stim-off meds-off, stim-on meds-off, and stim-on meds-on conditions. Additionally, all patients undergo automated gait and balance assessment in the same off and on medication conditions preoperatively and at all postoperative visits (in all conditions immediately after MDS-UPDRS part 3 scoring) using the APDM Mobility Lab [20] from which the iTUG and iSway data sets are obtained (S3 Appendix). Patients undergoing DBS for essential tremor or dystonia omit the PD-specific scales and undergo a videotaped Fahn-Tolosa-Marin tremor rating scale [21] or the Burke-Fahn-Marsden dystonia rating scale [22] respectively. Patients with prominent cervical dystonia are rated using the Toronto Western Spasmodic Torticollis Scale (TWSTRS) [23]. Cognitive function is tracked postoperatively using the Montreal Cognitive Assessment (MoCA) [24, 25]. All scores are entered into a REDCap database containing the elements shown in Table 1.

**Table 1:** Categories and descriptions of REDCap database forms.

Name of Data-entry form:	Type of data collected:
**Patient Information**	Patient names, physician names, and primary illness diagnosis (reasons for DBS)
**Demographics**	Gender, age, ethnicity, educational background, and marital status
**PD Features and Diagnosis**	Date of onset of motor symptoms, site of onset, medication usage, and presence of other symptoms related to PD
**PDQ-39**	Patient self-assessment of PD symptom severity pertaining to: mobility, daily living, emotional status, stigma, social support, cognitive impairment, communication, and bodily discomfort
**MDS UPDRS**	Investigator-rated assessment of cognitive and emotional symptoms, motor function, and severity of dyskinesia
**Quick Inventory of Depressive Symptomatology (QIDS)**	Patient self-assessment of mood, appetite and sleep
**Enhanced Speech and Swallowing Evaluation**	ENT-rated degree of speech and swallowing impairment.
**Neuropsychology**	Neuropsychology-rated degree of cognitive and emotional impairment.
**MINI-BEST**	Physical Therapy-assessed rating of patient balance and mobility
**TWSTRS**	Investigator-rated assessment of torticollis severity, including both pain and disability parameters
**Burke-Fahn-Marsden Dystonia Scale**	Investigator-rated assessment of dystonia type and severity
**Fahn-Tolosa-Marin Tremor Rating Scale**	Investigator-rated assessment of tremor in limbs, head, and trunk; also assesses handwriting, drawing, and water-pouring
**Global Surgical Outcomes**	Display of surgical results, possible lead implantation complications, and clinical impression rating by both physician and patient
**Mobility Lab**	Gait and balance assessment via APDM motion-tracking device
**Operative Record**	Surgical notes regarding target site for the lead, imaging modality usage, and MER recording details.
**Video Completion**	A “yes or no” placeholder indicating whether or not the patient’s assessment visit was recorded at that visit

PDQ-39 = Parkinson Disease Questionaire-39, MINI-BEST = Mini-Balance Evaluation Systems Test, TWSTRS = Toronto Western Spasmodic Torticollis Rating Scale.

### Cognitive testing

There is a growing body of literature suggesting that DBS can induce mood, cognitive or behavioral problems, and thus our approach has been to systematically evaluate each of these areas using preoperative neuropsychological testing. Since cognitive dysfunction occurs in many individuals with indications for DBS, some degree of cognitive impairment is not necessarily a contraindication for the procedure. Rather, the goals are to identify risk for major cognitive impairment or dementia and to establish a baseline for later comparison if needed to assess for change related to disease progression or surgical factors. Our evaluation includes assessment of mood, behavior, patient expectations for surgical outcome, and tolerance for undergoing a procedure that is performed awake. Through involvement of the family in the evaluation process, relevant psychosocial information is elicited that might have implications for DBS suitability. Results of cognitive testing also guide selection of the optimal surgical target. A list of the neuropsychological tests employed which take about 3 hours to administer, our method for determining T-scores, and an example of the display of cognitive data reviewed at the neuromodulation meeting is shown in S4 Appendix.

### Brain imaging

A single preoperative brain MRI is obtained to screen for the presence of contraindicating factors and to plan targeting for DBS lead implantation. Imaging is performed using a 32 channel transmit-receive head coil and a 3-Tesla horizontal bore MRI. On the day of the surgery, CT scans (1 mm slice thickness) with the patient’s head in the stereotactic frame are obtained and fused with preoperative MRI scans using the Framelink software (Medtronic Inc., Minneapolis, Mn.) to determine the stereotactic coordinates of the electrode. Following the completion of lead placement, postoperative CT scans are obtained looking for intracranial hemorrhage, edema and pneumocephalus. The postoperative CT scans are also fused with preoperative MRI scans to examine the position of each lead contact because knowledge of the actual position of each contact is useful when programming the neurostimulator. In the event of a surgical complication or where programming difficulty suggests lead malpositioning, a postoperative MRI is obtained on a 1.5 Tesla scanner. A protocol is utilized that provides good visualization of the lead position while maintaining head SAR below 0.1 W/Kg. Specifications of the MR imaging protocols is presented in S5 Appendix.

### Neuromodulation network meeting

The meeting utilizes a multidisciplinary approach to review patients being considered for DBS implantation to ensure optimal patient selection. At each monthly meeting the candidates eligible for surgery are reviewed in detail. The DBS coordinator presents a PowerPoint presentation of each candidate containing a summary of the history, list of current medications, results from prior medication trials and reasons for failure of other treatments, patient co-morbidities, an edited videotape of the off-on scoring visit, and the referring physician’s rationale for recommending DBS over other potential treatments (such as apomorphine or levodopa intestinal gel for PD fluctuators). The neuropsychologist presents results of cognitive testing, and representative members of the physical therapy and otolaryngology staff present results of balance and speech assessments. The neurosurgeon will comment on concerns (if any) related to possible operative complications based on patient co-morbidities. Discussion then ensues regarding the optimal target for implantation and what type of IPG would be most suitable for the particular patient. Any concerns about increased risks for a poor outcome based on patient characteristics are thoroughly explored to determine the risk-benefit ratio of DBS for that particular patient. This discussion is frequently supplemented by reference to high quality research studies in the field. Once a consensus is reached to either proceed with DBS or consider further medical treatment, a summary recommendation is documented in the patient’s medical chart, and the DBS coordinator notifies the patient of the plan for next steps.

### Surgical procedure

The details of the surgical procedure including our method of locating the surgical targets (STN, GPi, and VIM) are presented in S6 Appendix. For bilateral procedures, both leads are implanted in one surgical session, and the patient is brought back to the OR for implantation of the pulse generator one week later. In summary, the procedure begins with stereotactic localization of the target nucleus, burr holes are placed, microelectrode recording is performed to confirm accuracy of lead positioning, macrostimulation with the DBS lead is then performed with clinical observation of benefits and stimulation-related adverse effects. After the lead is secured, its position is checked with fluoroscopy and a postoperative CT scan is performed. The two major changes in our surgical procedure from implementation of PDSA cycles were the addition of microelectrode recording performed by a PhD neurophysiologist and the intentional targeting of trajectories to avoid passing through the lateral ventricles [26].

### Postoperative programming

The first programming session is typically scheduled 2–3 weeks after surgical implantation of the DBS electrodes to allow perioperative edema to resolve. A detailed evaluation of stimulation thresholds at each electrode using the battery (case) positive and electrode negative is performed which is often referred to as the “monopolar map”. Each electrode is tested to the maximally tolerated voltages (typically ranging from 4-5V) with a pulse width of 60–90 μs and frequency of 130 Hz [27]. The contact with the highest thresholds for stimulation side effects and favorable clinical effects is selected utilizing moderate amplitude (2–2.5V) with standard pulse width and frequencies. Often improvement of tremor is observed in clinic, but for dystonia and other PD symptoms there is often a delayed benefit observed a week to several months after initial setting of stimulation parameters. A second programming visit is scheduled approximately 6–8 weeks after activating the device in order to evaluate the initial response to stimulation and to make any necessary adjustments. When there is poor symptom control with monopolar settings additional contacts can be added to create a double monopolar program. If there are intolerable side effects at settings that otherwise improve symptoms, the field of stimulation can be reduced by using bipolar stimulation (an electrode is set as positive). Finally, interleaved programming allows for rapid alternating delivery of stimulation through 2 separate contacts which can be used when there is difficulty obtaining adequate symptom control at a single contact [28].

### Outcomes review meeting

Once our database and neuromodulation network was fully functioning, we began having regular outcomes review meetings to assess the quality of patient outcomes and to consider ways to address any problems noted. At these meetings the results of motor, cognitive and quality of life data are reviewed, along with any surgical complications. Patients not receiving adequate clinical improvement following surgery have their cases analyzed in detail to determine the cause of the problem (patient selection, lead positioning, or programming difficulties). Findings from these meetings are used to fuel additional PDSA cycles aimed at further improving the quality of our program.

### Preliminary outcomes data

**Fig 1:**
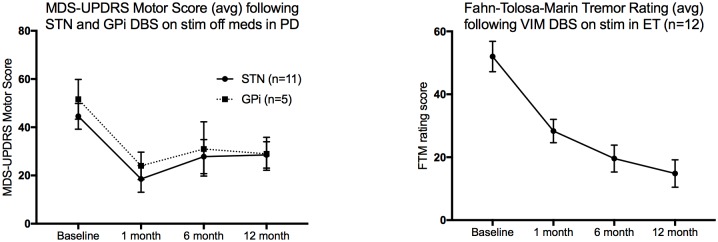
Motor outcomes in PD and ET patients since initiation of the neuromodulation network and REDCap database outcomes tracking. Error bars represent SEM.

Fig 1 shows motor outcomes in our PD and ET subjects who have undergone surgery since the development of our neuromodulation database. Table 2 lists surgical complications encountered.

**Table 2:** Surgical complications.

	Dx	Age (yrs)	Sex	Target	Surgery-related Complication	Device-related Complication
Case 1	PD	51	M	STN	Lead infections and erosion of leads through scalp	
Case 2	PD	73	F	STN	Post-op seizure, edema near left DBS lead	
Case 3	PD	54	M	STN	Post-op speech deficits and falls	
Case 4	PD	76	M	STN	Small track hemorrhage around right DBS lead with transient delirium, disorientation, visual and auditory hallucinations	
Case 5	PD	53	M	STN		Skin breakdown/erosion of hardware
Case 6	ET	71	F	VIM		IPG wound required surgical revision
Case 7	ET	81	M	VIM	Transient confusion, IPG infection	Continuing unexplained shocks during stimulation
Case 8	ET	72	M	VIM	Cyst developed around right lead	
Case 9	ET	58	M	VIM	IPG infection, resolved	

Dx = diagnosis, PD = Parkinson disease, ET = essential tremor, STN = subthalamic nucleus, VIM = ventrointermedius nucleus of the thalamus.

## Discussion

We have instituted a continuous improvement process in our DBS program using PDSA methodology for the overall program as well as each of the key components of the program. This is an outcomes-driven process, and input from a broad range of professionals has been welcomed in making iterative changes in the way we select patients for surgery, implant the leads, and program the stimulator in order to provide greatest benefits to patients considering the major undertaking of stereotactic neurosurgery. The circumstances in any individual hospital will be unique, but we hope the continuous improvement process described here can help other DBS programs achieve and demonstrate the best outcomes possible. The systematic steps described here constituted a change in culture for physicians who were accustomed to making expert decisions based on individual knowledge of their patients in the context of the physician’s own experience with DBS. Consistent and skilled leadership was vital to keeping the stakeholders on board with the process. We also encountered a financial challenge: we had to justify to the hospital why it was in their interest to support the costs of implementing improvement steps including new software, hardware, and personnel. This has been occurring at a time when margins are tight and when payment for surgery is not impacted by surgical outcomes. We are grateful the financial decision-makers supported this physician-led initiative, and we hope the hospital accrues benefits including financial return on their investment as payment models move increasingly toward value in the coming years.

Limitations of our QI process and this report include the absence of tracking of outcomes of patients who enter the pathway who do not undergo surgery—we would like to know that our decision to recommend against surgery following our protocol turns out to be a good decision for those patients also—however it is difficult to track outcomes for these patients especially if they transfer care elsewhere. In addition, the goals and targets for improvement are somewhat arbitrary, though guided by literature review. Additionally, the triggers for making a change in a protocol are somewhat fuzzy—when new technology became available, or when new information was added to the published literature this has sometimes resulted in a revision in our protocols and sometimes not. In spite of greatest attempts to hardwire an algorithm into the decision tree, there is considerable room for expert opinion, perhaps inevitable in the technologically evolving and complex clinical field that is DBS therapy. Finally, a fundamental limitation of this report is that because we did not track outcomes prior to initiation of this QI project, we cannot prove that our results are better than before this project was initiated.

Further comment is needed on the issue of costs involved in instituting a neuromodulation pathway. Our practice is to bill payers or patients for the costs of all pre-operative consultations and imaging tests, as these are required to make decisions on the likely safety and efficacy of the planned procedure. However, we do not consider it justified to bill third party payers or patients for repeating many of these measures postoperatively purely to track outcomes, because outcome tracking in some of these areas does not directly benefit an individual patient nor lead to change in the treatment plan. Examples of pre-operative tests which we do not routinely perform postoperatively include neuropsychological testing, speech, swallowing and balance assessments, and MR imaging. In order to repeat any of these studies, there would need to be a clinical indication for obtaining these repeat tests such as a surgical complication, or a patient who fails to respond as expected to this therapy. On the other hand, detailed motor scoring and quality of life assessments must be done both pre- and at defined intervals post-operatively in order measure the effectiveness and safety of the overall program, yet the costs of these are not billable to patients or third parties. Accordingly, support from the hospital system or academic department is needed to provide this important monitoring function. At our institution, we have a full-time DBS coordinator who is skilled in administration of motor and quality of life scales who schedules ongoing post-operative scoring visits at no charge to patients or payers.

As shown in our preliminary outcomes data, we did experience 9 surgical or device-related complications for procedures performed in a total of 28 patients. While this appears to represent a high complication rate, it is important to note that we captured all complications, however minor or transient, which is rarely the case in published studies. Our neuromodulation network ensures that minor surgical complications will be identified, and when trends are noted which are amenable to a PDSA improvement cycle, it can be implemented without delay.

Our future plans include indefinite continuation of the neuromodulation network with regular outcome analyses to inform additional quality improvement initiatives. Once we have a significant number of post-operative MRI scans in patients with suboptimal outcomes, we will examine lead location and try to correlate this with clinical data and surgical details to design a PDSA cycle to further improve lead localization. Through ongoing data collection of long-term motor and quality of life outcomes, we hope to further improve the overall safety and efficacy of DBS surgery for movement disorders.

## Supporting Information

S1 Appendix Neuromodulation patient handouts and checklist. (DOCX)

S2 Appendix Clinical global impression scale. (DOCX)

S3 Appendix Description of APDM Mobility Lab. (DOCX)

S4 Appendix Description of neuropsychological testing. (DOCX)

S5 Appendix MR imaging specifications. (DOCX)

S6 Appendix Description of surgical procedure. (DOCX)
