# Supplementary material for: Developing a Deep Brain Stimulation Neuromodulation Network for Parkinson Disease, Essential Tremor, and Dystonia: Report of a Quality Improvement Project
Source: PLoS One. 2016 Oct 6;11(10):e0164154. doi: 10.1371/journal.pone.0164154 (PMC5053513; doi:10.1371/journal.pone.0164154)
Supplement: S1 Appendix — (DOCX) [file pone.0164154.s001.docx]

**S1 Appendix**

**DBS Neuromodulation pathway CHECKLIST**

There are several steps that you need to complete to determine if you would be a good candidate for DBS surgery. Your neurologist will place all the necessary referrals. It is your responsibility to respond to scheduling requests in a timely manner and attend all necessary visits. It may take about 3 months to complete all the visits. Please contact neurology clinic if you have any questions.

**Neurocognitive testing**

**MRI brain**

**Physical therapy (PT) pre-op assessment**

**ENT and speech therapy (ST) pre-op assessment**

**Exam video recording and questionnaires** this is a one-time visit to document your symptoms on video. Instructions for video visit: you will be scheduled for a 2 hour visit in the morning. You need to come off medications meaning no Parkinson’s medications after midnight. Do not take any long acting Parkinson’s medications (Requip XL or Mirapex ER) the day before the visit, and remove Neupro patch the evening before the visit. You need to come on an empty stomach (no food, but coffee, water or juice are fine). Please bring all your Parkinson’s medication bottles, and a bottle of your favorite soda (fizzy) drink which will speed up levodopa absorption when we give it in the clinic.

**Please call the neurology clinic to let us know when you have completed all the steps, or if the process seems to have stalled at any point.**

After the checklist is completed, your case will be presented at the **Neuromodulation meeting** to determine if you would be a good candidate for DBS surgery. Meetings are conducted once a month. You will be notified of the committee’s recommendation within 1 week after your case is presented. If you are deemed to be a good candidate, you will receive an appointment date with our neurosurgeon for a **pre-op neurosurgery clinic visit.** Once cleared by the surgeon, you will receive a surgery date. You will also be invited to attend an optional **DBS educational session** provided by Medtronic (the manufacturer of DBS hardware). Due to a waiting list, your surgery date may be several months from your initial evaluation. You will be asked to come in for a **pre-op neurology clinic visit** within 2 weeks before the surgery. If you live far away, this visit may in some cases be done over the phone. The initial DBS **programming visit** will be scheduled 3-4 weeks after the surgery to allow your brain to recover.

**Deep Brain Stimulation For Parkinson Disease**

This handout is to provide information to help you decide whether to proceed with bilateral deep brain stimulation (DBS) for Parkinson disease (PD). Please consider this carefully and discuss any questions you have about these points with your doctor before proceeding with brain surgery.

**WHAT IS DBS?** DBS involves electrical stimulation of nerves in the part of the brain that are involved in controlling movement. The electrical current is generated by a battery pack that is implanted beneath the skin, usually underneath the collarbone. The current gets to the appropriate brain site by way of a thin electrode that is implanted through a hole in the skull.

DBS has been practiced for over 20 years. Placing the wire in the appropriate location of the brain and stimulating these regions has been increasingly practiced the United States since the late 1990s. In general it is felt to be the most effective currently approved surgery for PD.

**WHAT WILL IT DO?** DBS modulates electrical activity in selected areas of the brain leading to improvement of certain symptoms of PD. Typically tremor, slowness of movement, and rigidity improve to a degree after surgery. The surgery often will allow for a reduction of your medication dose and potentially alleviate troubling dyskinesias resulting from levodopa (Sinemet). Additionally, motor fluctuations caused by medication wearing off can be reduced with DBS. As with most forms of brain surgery, the effects are most prominent on the side of the body OPPOSITE to the side of stimulation. Since most PD patients have symptoms on both sides of the body, we generally recommend placement of two stimulators, one on each side of the brain.

**WHAT WON’T IT DO?** This treatment is not curative. Regardless of any initial improvement, your parkinsonian symptoms will worsen from year to year after surgery as the underlying disease progresses. To the best of our knowledge, the brain cells affected by PD continue to deteriorate at about the same rate whether or not you undergo this treatment. **It is important to remember that the effects of DBS are very similar to the effects of levodopa and therefore if a certain symptom you have does NOT get better following a dose of levodopa, it is unlikely to improve with DBS with the exception of tremor.** Balance and speech are common problems in this category that may not improve at all following this procedure.

DBS does not treat non-motor symptoms such as sleep, memory problems, dizziness, mood disorders, and bladder and bowel dysfunction which are commonly affected in PD.

**WILL MY MEDICATION CHANGE AFTERWARDS?** Your doctor may reduce doses of medication, for example Sinemet, in the months after surgery, particularly if the surgery has a good effect on your symptoms. It is not likely you will be able to stop PD medications altogether.

**DOES EVERYONE WITH PD QUALIFY FOR DBS?** No. To assess your fitness for surgery, your treating Neurologist at UT Southwestern will initiate a mandatory multidisciplinary evaluation encompassing several steps over a period of time involving motor scoring, cognitive testing, physical and speech therapy evaluations, neurosurgical consultation, and a brain MRI. If you have general medical conditions that increase your risk for surgical complications, the neurosurgeon may suggest additional testing. Data from these assessments will be collected and reviewed at a monthly conference, attended by the neurologists, the neurosurgeon and rehabilitation and neurocognitive services, where your final candidacy for DBS will be decided. The entire process, called the Neuromodulation Pathway, from your initial evaluation to DBS surgery may take several months.

**WHAT DOES DBS SURGERY INVOLVE? WHAT ARE THE POTENTIAL RISKS?** The neurosurgeon and his associates will discuss this with you in more detail. Briefly, infections, brain hemorrhage, and stroke are the most serious potential risks. DBS surgery is done in two stages a week apart. During Stage I, electrodes are implanted in the appropriate brain targets and after 24 hours of monitoring you are released home. You are off your PD medications for at least 12 hours and mostly awake during surgery to ensure the electrode is in the right spot by being examined for improvement of tremor, stiffness and slowness as the wires are advanced into the brain. Microelectrode recordings are used to help identify correct location for electrode implantation. Electrical stimulation of the brain targets is done to test for improvement of motor symptoms and to look for thresholds at which side effects might occur. At Stage II, the Implantable Pulse Generator (sometimes called the battery pack) is inserted in your chest wall and connected to the brain electrodes.

**WHAT HAPPENS AFTER DBS SURGERY?** About 1 month after surgery, you will be evaluated by your neurologist for programming of the DBS electrodes, a process that takes a few hours. As part of the ongoing Neuromodulation Pathway, you will be asked to return for DBS programming and clinical scoring at regular intervals. These visits will occur in the morning, continue through the lunch hour, and involve video recording and careful motor scoring. You should hold your medications for at least 8 hours before each programming appointment but bring them to the clinic as a portion of the evaluation also involves testing you after you have taken your medication. Regular DBS programming is a prerequisite to the improvement of your motor symptoms.

**WHAT BENEFITS CAN I REALISTICALLY EXPECT?** The goal is to accomplish the benefits of levodopa, with less of the drug side effects, and in a more sustained fashion than can be accomplished with periodic oral dosing. The benefits of this treatment may take months to achieve. The magnitude of the benefit in a particular case is not possible to predict. In some cases the benefits are quite modest. In occasional cases, benefits are non-existent. The best response that can realistically be hoped for is the response that you typically achieve during a good response to medications with fewer or absent dyskinesias and more consistent on time.

**WILL THE OPERATION AFFECT MY THINKING?** Mental abilities can be worsened by the surgery. This can be temporary or permanent. It is widely believed, though as yet unproven, that patients with problems with memory and thinking are at greatest risk for deterioration after surgery. If results of the neurocognitive test are below normal, the risk of mental worsening rises and the chances of a good physical outcome may fall. It is commonly felt that older patients have a greater risk than younger patients of mental deterioration after the treatment.

**WILL THERE BE ANY LIMITATIONS IN ACCESS TO MEDICAL PROCEDURES AFTER THIS PROCEDURE?** At the present time, many centers will refuse to perform MRI scans on patients who have DBS stimulators due to a possible safety concern. Limited MRI of the brain may be safely performed, but only at specialized DBS centers such as ours. Additionally, patients should never undergo a procedure called “diathermy” offered by some dentists for the treatment of some tooth disorders.

**ADDITIONAL COMMENTS:**

______________________________________________________________________________________________________________________________________________________________________________________________________________________________________________________________________________________________________________________________________________________________________________________________________________________________________________________________________________________________________________________________________________________________________________________________________________________________________________________________________________________________________________________________________________________________________________________________________________________________________________________________________________________________________________________________________________________________________________________________________________________________________________________________

If you have read and understood the above facts, please sign below to indicate your receipt of and understanding of this informational handout.

____________________________________________________________________________________________

Patient’s Name (print) Signature Date

**Deep Brain Stimulation For Essential Tremor**

This handout is to provide information to help you decide whether to proceed with bilateral deep brain stimulation (DBS) for essential tremor (ET). Please consider this carefully and discuss any questions you have about these points with your doctor before proceeding with brain surgery.

**WHAT IS DBS?** DBS involves electrical stimulation of nerves in the part of the brain that are involved in controlling movement. The electrical current is generated by a battery pack that is implanted beneath the skin, usually underneath the collarbone. The current gets to the appropriate brain site by way of a thin electrode that is implanted through a hole in the skull.

DBS has been practiced for over 20 years. Placing the wire in the appropriate location of the brain and stimulating these regions has been increasingly practiced the United States since the late 1990s. In general it is felt to be the most effective currently approved surgery for ET.

**WHAT WILL IT DO?** DBS modulates electrical activity in selected areas of the brain leading to improvement of tremor. Tremor may not be completely eliminated, but with correctly positioned electrodes, it should be significantly improved (on the order of 50-80%). As with most forms of brain surgery, the effects are most prominent on the side of the body OPPOSITE to the side of stimulation. Since most ET patients have symptoms on both sides of the body, we generally recommend placement of two electrodes, one on each side of the brain. If your tremor is bothersome on only one side of your body, we may recommend the placement of only one electrode on the side of the brain opposite your most troublesome tremor. Placement of only one electrode may result in fewer side effects.

**WHAT WON’T IT DO?** This treatment is not curative. Regardless of any initial improvement, your tremor may worsen from year to year after surgery. Periodic adjustment of stimulation parameters may provide additional benefit. Voice tremor may not improve after the procedure. Speech and balance may worsen after the procedure, but these side effects can usually be managed by stimulation adjustment (change in the amount of electrical current delivered to the brain). In some ET patients, balance may progressively worsen regardless of the stimulation as part of the underlying disease

**WILL MY MEDICATION CHANGE AFTERWARDS?** Your doctor may reduce doses of medication after surgery, particularly if the surgery has a good effect on your symptoms. Many patients are able to stop anti-tremor medications altogether.

**DOES EVERYONE WITH ET QUALIFY FOR DBS?** No. To assess your fitness for surgery, your treating Neurologist at UT Southwestern will initiate a mandatory multidisciplinary evaluation encompassing several steps over a period of time involving motor scoring, cognitive testing, physical and speech therapy evaluations, neurosurgical consultation, and a brain MRI. If you have general medical conditions that increase your risk for surgical complications, the neurosurgeon may suggest additional testing. Data from these assessments will be collected and reviewed at a monthly conference, attended by the neurologists, the neurosurgeon and rehabilitation and neurocognitive services, where your final candidacy for DBS will be decided. The entire process, called the Neuromodulation Pathway, from your initial evaluation to DBS surgery may take several months.

**WHAT DOES DBS SURGERY INVOLVE? WHAT ARE THE POTENTIAL RISKS?** The neurosurgeon and his associates will discuss this with you in more detail. Briefly, infections, brain hemorrhage, and stroke are the most serious potential risks. DBS surgery is done in two stages a week apart. During Stage I, electrodes are implanted in the appropriate brain targets and after 24 hours of monitoring you are released home. You are mostly awake during surgery to ensure the electrode is in the right spot by being examined for improvement of tremor as the wires are advanced into the brain. Electrical stimulation of the brain targets is done to test for improvement of tremor and to look for thresholds at which side effects might occur. At Stage II, the Implantable Pulse Generator (sometimes called the battery pack) is inserted in your chest wall and connected to the brain electrodes, after which you will be monitored for several hours and released home on the same day.

**WHAT HAPPENS AFTER DBS SURGERY?** About 1 month after surgery, you will be evaluated by your neurologist for programming of the DBS electrodes, a process that takes a few hours. As part of the ongoing Neuromodulation Pathway, you will be asked to return for DBS programming and clinical scoring at regular intervals. These visits will occur in the morning, continue through the lunch hour, and involve video recording and careful tremor scoring. Regular DBS programming is a prerequisite to the improvement of your tremor.

**WHAT BENEFITS CAN I REALISTICALLY EXPECT?** The goal is to reduce your tremor as much as possible without causing stimulation-related side effects. The benefits of this treatment may take months to achieve. The magnitude of the benefit in a particular case is not possible to predict. In some cases the benefits are quite modest. In occasional cases, benefits are non-existent. In some cases, in order to minimize stimulation-related side effects, the device may be turned down on the side opposite the non-dominant limb resulting in less-than-optimal tremor control on that side of the body.

**WILL THE OPERATION AFFECT MY THINKING?** Mental abilities can be worsened by the surgery. This can be temporary or permanent. It is widely believed, though as yet unproven, that patients with problems with memory and thinking are at greatest risk for deterioration after surgery. If results of the neurocognitive test are below normal, the risk of mental worsening rises and the chances of a good physical outcome may fall. It is commonly felt that older patients have a greater risk than younger patients of mental deterioration after the treatment.

**WILL THERE BE ANY LIMITATIONS IN ACCESS TO MEDICAL PROCEDURES AFTER THIS PROCEDURE?** At the present time, many centers will refuse to perform MRI scans on patients who have DBS stimulators due to a possible safety concern. Limited MRI of the brain may be safely performed, but only at specialized DBS centers such as ours. Additionally, patients should never undergo a procedure called “diathermy” offered by some dentists for the treatment of some tooth disorders.

**ADDITIONAL COMMENTS:**

______________________________________________________________________________________________________________________________________________________________________________________________________________________________________________________________________________________________________________________________________________________________________________________________________________________________________________________________________________________________________________________________________________________________________________________________________________________________________________________________________________________________________________________________________________________________________________________________________________________________________________________________________________________________________________________________________________________________________________________________________________________________________________________________

If you have read and understood the above facts, please sign below to indicate your receipt of and understanding of this informational handout.

____________________________________________________________________________________________

Patient’s Name (print) Signature Date

**Deep Brain Stimulation For Dystonia**

This handout is to provide information to help you decide whether to proceed with bilateral deep brain stimulation (DBS) for dystonia. Please consider this carefully and discuss any questions you have about these points with your doctor before proceeding with brain surgery.

**WHAT IS DBS?** DBS involves electrical stimulation of nerves in the part of the brain that are involved in controlling movement. The electrical current is generated by a battery pack that is implanted beneath the skin, usually underneath the collarbone. The current gets to the appropriate brain site by way of a thin electrode that is implanted through a hole in the skull.

DBS has been practiced for over 20 years. Placing the wire in the appropriate location of the brain and stimulating these regions has been increasingly practiced the United States since the late 1990s. In general it is felt to be the most effective currently approved surgery for dystonia.

**WHAT WILL IT DO?** DBS modulates electrical activity in selected areas of the brain leading to improvement of certain symptoms of dystonia. Typically abnormal muscle contractions improve to a degree after surgery. The surgery typically will allow for a reduction of your medication dose. As with most forms of brain surgery, the effects are most prominent on the side of the body OPPOSITE to the side of stimulation. Since most dystonia patients have symptoms on both sides of the body, we generally recommend placement of two electrodes, one on each side of the brain.

**WHAT WON’T IT DO?** This treatment is not curative. Regardless of any initial improvement, your dystonia symptoms may worsen from year to year after surgery. Periodic adjustment of stimulation parameters may provide additional benefit.

DBS does not treat non-motor symptoms such as sleep, memory problems, dizziness, mood disorders, and bladder and bowel dysfunction.

**WILL MY MEDICATION CHANGE AFTERWARDS?** Your doctor may reduce doses of medication after surgery, particularly if the surgery has a good effect on your symptoms. Some patients are able to stop anti-dystonia medications altogether.

**DOES EVERYONE WITH DYSTONIA QUALIFY FOR DBS?** No. To assess your fitness for surgery, your treating Neurologist at UT Southwestern will initiate a mandatory multidisciplinary evaluation encompassing several steps over a period of time involving motor scoring, cognitive testing, physical and speech therapy evaluations, neurosurgical consultation, and a brain MRI. If you have general medical conditions that increase your risk for surgical complications, the neurosurgeon may suggest additional testing. Data from these assessments will be collected and reviewed at a monthly conference, attended by the neurologists, the neurosurgeon and rehabilitation and neurocognitive services, where your final candidacy for DBS will be decided. The entire process, called the Neuromodulation Pathway, from your initial evaluation to DBS surgery may take several months.

**WHAT DOES DBS SURGERY INVOLVE? WHAT ARE THE POTENTIAL RISKS?** The neurosurgeon and his associates will discuss this with you in more detail. Briefly, infections, brain hemorrhage, and stroke are the most serious potential risks. DBS surgery is done in two stages a week apart. During Stage I, electrodes are implanted in the appropriate brain targets and after 24 hours of monitoring you are released home. You are mostly awake during surgery to ensure the electrode is in the right spot by monitoring brain activity with microelectrode recordings. Electrical stimulation of the brain targets is done to test for improvement of motor symptoms and to look for thresholds at which side effects might occur. At Stage II, the Implantable Pulse Generator (sometimes called the battery pack) is inserted in your chest wall and connected to the brain electrodes, after which you will be monitored for several hours and released home on the same day.

**WHAT HAPPENS AFTER DBS SURGERY?** About 1 month after surgery, you will be evaluated by your neurologist for programming of the DBS electrodes, a process that takes a few hours. As part of the ongoing Neuromodulation Pathway, you will be asked to return for DBS programming and clinical scoring at regular intervals. These visits will occur in the morning, continue through the lunch hour, and involve video recording and careful motor scoring. Regular DBS programming is a prerequisite to the improvement of your motor symptoms. It is crucial to remember that dystonia symptoms take MONTHS to improve after the surgery.

**WHAT BENEFITS CAN I REALISTICALLY EXPECT?** The goal is to improve dystonia motor symptoms. The benefits of this treatment will take months to achieve. The magnitude of the benefit in a particular case is not possible to predict. In some cases the benefits are quite modest. In occasional cases, benefits are non-existent. Scientific literature cites improvements typically between 25%-80%. Outcome will also depend on the type of dystonia that you have (primary vs. secondary; generalized vs. focal), the age at which you are having surgery, and how long you have suffered from dystonia before undergoing surgery. You should discuss these issues in detail with your neurologist.

**WILL THE OPERATION AFFECT MY THINKING?** Mental abilities can be worsened by the surgery. This can be temporary or permanent. It is widely believed, though as yet unproven, that patients with problems with memory and thinking are at greatest risk for deterioration after surgery. If results of the neurocognitive test are below normal, the risk of mental worsening rises and the chances of a good physical outcome may fall. It is commonly felt that older patients have a greater risk than younger patients of mental deterioration after the treatment.

**WILL THERE BE ANY LIMITATIONS IN ACCESS TO MEDICAL PROCEDURES AFTER THIS PROCEDURE?** At the present time, many centers will refuse to perform MRI scans on patients who have DBS stimulators due to a possible safety concern. Limited MRI of the brain may be safely performed, but only at specialized DBS centers such as ours. Additionally, patients should never undergo a procedure called “diathermy” offered by some dentists for the treatment of some tooth disorders.

**ADDITIONAL COMMENTS:**

______________________________________________________________________________________________________________________________________________________________________________________________________________________________________________________________________________________________________________________________________________________________________________________________________________________________________________________________________________________________________________________________________________________________________________________________________________________________________________________________________________________________________________________________________________________________________________________________________________________________________________________________________________________________________________________________________________________________________________________________________________________________________________________________

If you have read and understood the above facts, please sign below to indicate your receipt of and understanding of this informational handout.

____________________________________________________________________________________________

Patient’s Name (print) Signature Date
