# Supplementary material for: Developing a Deep Brain Stimulation Neuromodulation Network for Parkinson Disease, Essential Tremor, and Dystonia: Report of a Quality Improvement Project
Source: PLoS One. 2016 Oct 6;11(10):e0164154. doi: 10.1371/journal.pone.0164154 (PMC5053513; doi:10.1371/journal.pone.0164154)
Supplement: S2 Appendix — (DOCX) [file pone.0164154.s002.docx]

**S2 Appendix**

CLINICAL GLOBAL IMPRESSION (CGI) SCALE

***To be completed by patient AND programming neurologist at each post-op visit***

-5 very poor outcome with marked constant worsening of symptoms, with or without appearance of stimulation induced symptoms impacting quality of life (e.g. dysarthria, gait problems)

-4 poor outcome with severe constant worsening of symptoms with or without appearance of stimulation induced symptoms impacting quality of life (e.g. dysarthria, gait problems)

-3 poor outcome with moderate constant worsening of symptoms or severe intermittent worsening of symptoms with or without appearance of stimulation induced symptoms impacting quality of life (e.g. dysarthria, gait problems)

-2 poor outcome with mild constant worsening of symptoms or moderate intermittent worsening of symptoms with or without appearance of stimulation induced symptoms impacting quality of life (e.g. dysarthria, gait problems)

-1 poor outcome with mild intermittent worsening of symptoms with or without appearance of stimulation induced symptoms impacting quality of life (e.g. dysarthria, gait problems)

0 no change from pre-surgical level

+1 intermittent improvement, with or without tolerable side effects

+2 mild constant improvement, with or without tolerable side effects

+3 moderate constant improvement with no side effects or very mild intermittent side effects

+4 High level of constant improvement with no side effects

+5 Marked constant improvement with no side effects, all expectations are met, best possible outcome achieved

***NOTE***: All answers 0 through -5 will be associated with the rater (physician and/or patient) regretting that they underwent DBS surgery. If the patient does NOT regret surgery, by definition they are doing at least ***some*** better than baseline. So always ask them if they regret having had the procedure, and if not, this gets you at least into positive Likert territory.
