# Supplementary material for: Developing a Deep Brain Stimulation Neuromodulation Network for Parkinson Disease, Essential Tremor, and Dystonia: Report of a Quality Improvement Project
Source: PLoS One. 2016 Oct 6;11(10):e0164154. doi: 10.1371/journal.pone.0164154 (PMC5053513; doi:10.1371/journal.pone.0164154)
Supplement: S3 Appendix — (DOCX) [file pone.0164154.s003.docx]

**S3 Appendix**

**APDM Mobility Lab**

The Mobility Lab™ from APDM, Inc. (<http://apdm.com/products/software/mobilitylab/>) is a portable computerized measurement system which uses up to 6 inertial wireless sensors to quantify gait and balance. The sensors employ triaxial accelerometers, gyroscopes, and magnetometers to measure kinetic parameters during predefined tasks such as the timed up and go test, postural sway in quiet stance test, and walking. Data generated by these sensors include iTUG and iSWAY (instrumented timed up and go test and body sway test), a collection of quantifiable variables that can be analyzed and compared between study subjects and normal controls. Components of the Mobility Lab™ are illustrated below.


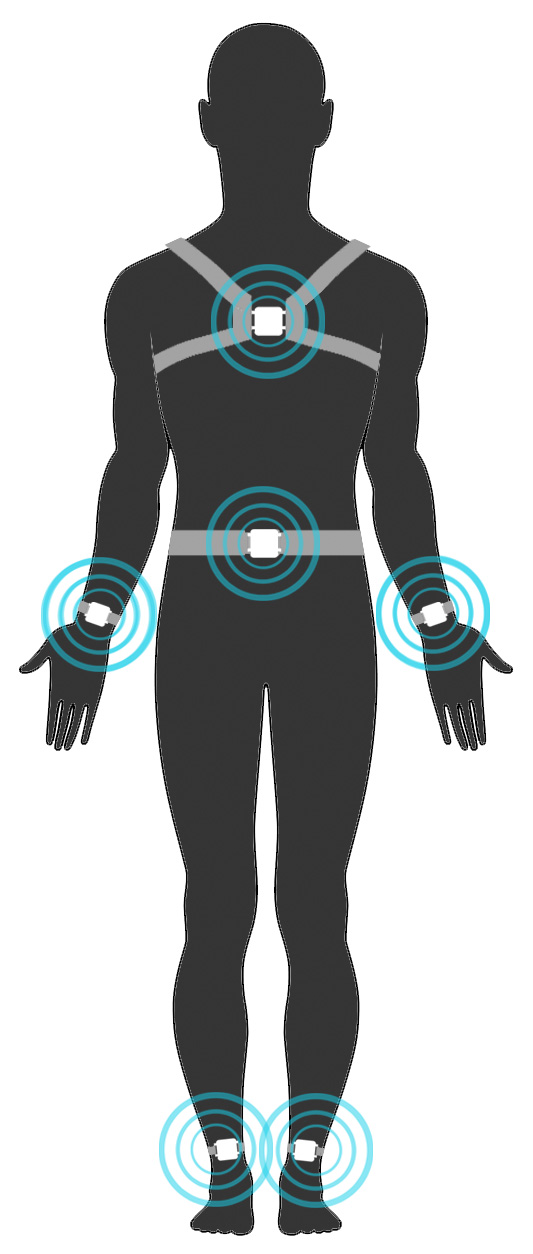
 **Computer and receiver Wireless sensor Sensors attached to subject**


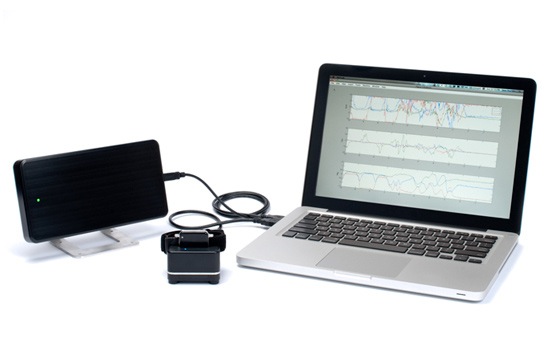

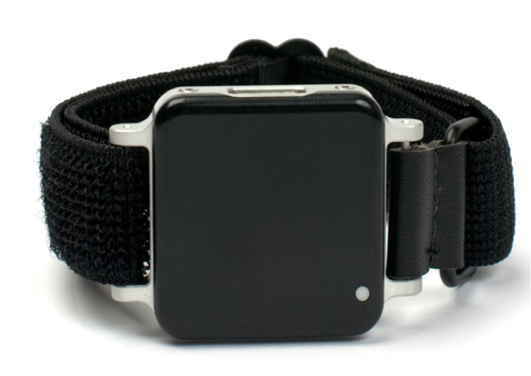


The iTUG plugin processes input signals and provides objective measures of gait: turning, gait, sit-to-stand, and turn-to-sit. iSWAY measures sway characteristics in both the time and frequency domains. These programs are illustrated here:


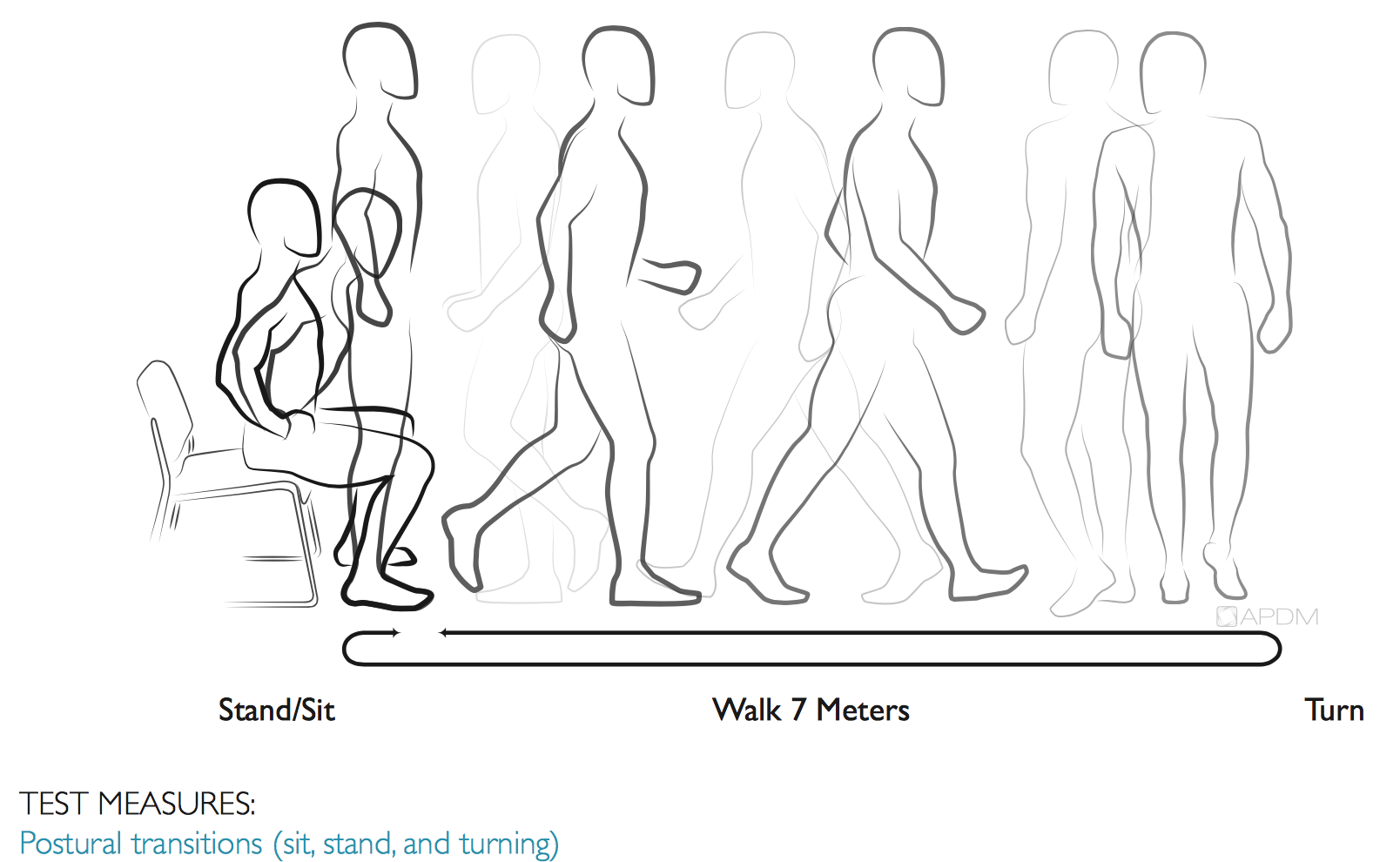
iTUG:


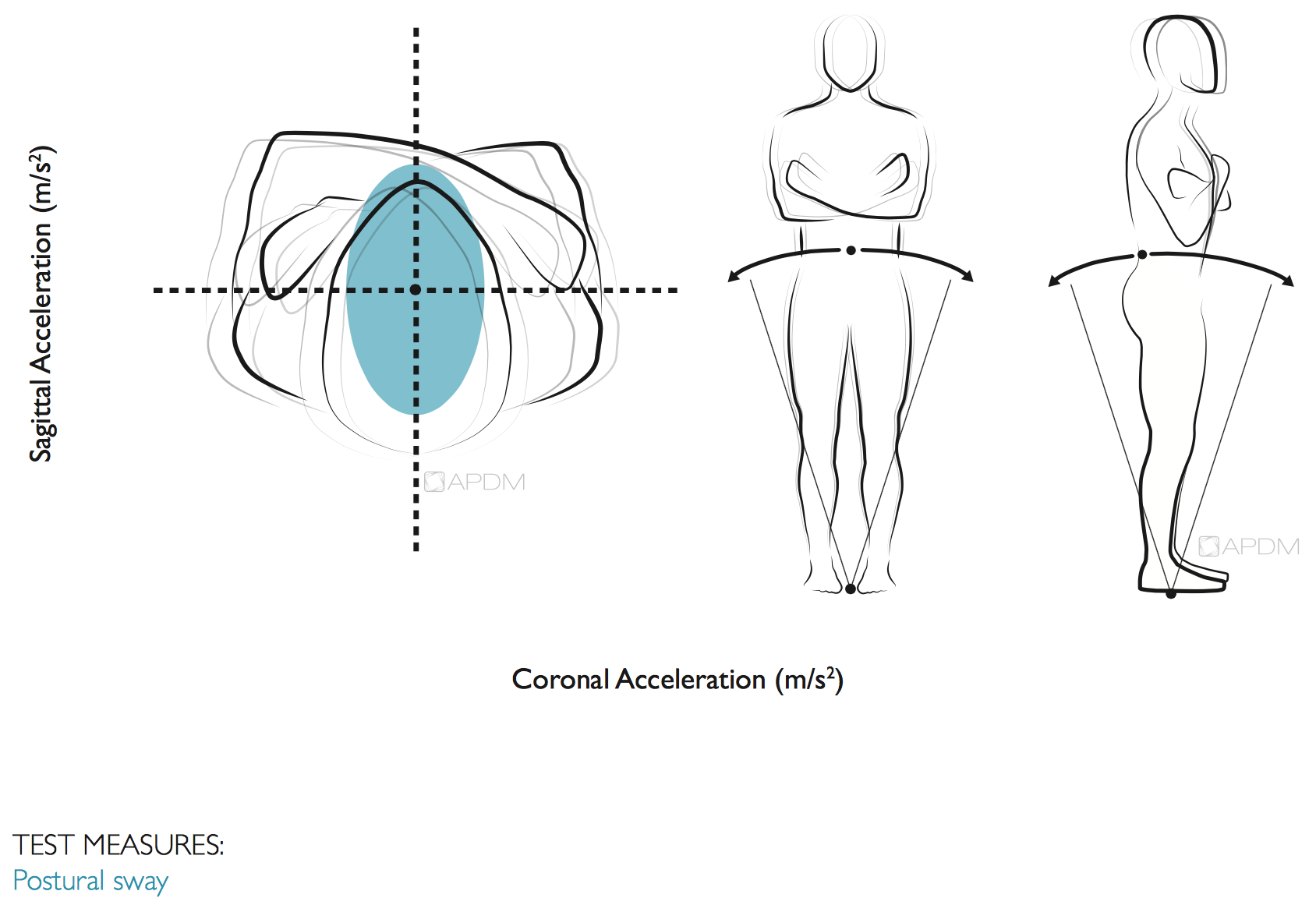
iSway:
