# Supplementary material for: Developing a Deep Brain Stimulation Neuromodulation Network for Parkinson Disease, Essential Tremor, and Dystonia: Report of a Quality Improvement Project
Source: PLoS One. 2016 Oct 6;11(10):e0164154. doi: 10.1371/journal.pone.0164154 (PMC5053513; doi:10.1371/journal.pone.0164154)
Supplement: S4 Appendix — (DOCX) [file pone.0164154.s004.docx]

**S4 Appendix**

**Neuropsychological Testing**

A list of our neuropsychological test battery is shown in Table 1. Note that alternative tests are used in instances where the patient’s movement difficulties are thought to interfere with or prohibit task completion. Embedded measures of effort are examined (i.e., Reliable Digit Span, CVLT-II Forced Choice) to assess confidence in the results. The battery takes approximately 2.5 to 3 hours to complete and the order of tests is varied depending on patient “on/off” states to maximize performance. Key scores from each test are converted to T scores using demographically corrected normative data and then used to derive cognitive domain T scores as well as a global cognitive T score (Figure 1). These variables are all entered into the REDCap database along with the individual test scores. The domain T scores are further used along with qualitative analysis of test performance by the neuropsychologist to assign a clinical rating of performance for each cognitive domain, which is then displayed in graph format (Figure 2). The graph allows for easy and clear review of cognitive performance at the neuromodulation meeting.

**Table 1. Neuropsychological Battery for Pre-DBS Evaluation**

| **General** |
| --- |
| Wechsler Adult Intelligence Scale-IV (WAIS-IV; Vocabulary + Block Design) |
| Test of Premorbid Functioning (Pre-morbid IQ estimate) |
| Dementia Rating Scale-2 |
| **Executive Functioning** |
| Wisconsin Card Sorting Test |
| Trail Making Test Part B |
| FAS Verbal Fluency |
| **Attention/Processing Speed** |
| Trail Making Test Part A |
| WAIS-IV Digit Span and Digit Symbol Coding or Symbol Search* |
| **Language** |
| Boston Naming Test |
| Animal Fluency |
| **Visuospatial** |
| WAIS-IV Block Design or Matrix Reasoning* |
| Clock Drawing |
| Rey-Osterrieth Complex Figure Copy |
| **Memory** |
| California Verbal Learning Test - II |
| Rey-Osterrieth Complex Figure or Warrington Recognition Memory Test (Faces)* |
| **Mood/Behavior** |
| Beck Depression Inventory-II |
| Questionnaire for Impulsive-Compulsive Disorders in Parkinson’s Disease – Rating Scale (QUIP-RS) |

*Alternate test if needed due to motor difficulties

Figure 1. Cognitive domain T scores are derived by averaging T scores for selected cognitive variables within each domain. A global T score is then derived by average the domain T scores.

Figure 2. The graph below represents clinical ratings for each cognitive domain assessed, including an index of depression that takes into account qualitative aspects of the patient's performance as well as standard scores. In this instance, the patient demonstrates global impairment with greatest difficulty in nonverbal memory and attention, and some declines in executive functioning, language, and visuospatial functioning, raising concerns for presence of a dementia.

Est IQ = Estimated IQ, Exec Fx = Executive Functioning, Attn = Attention, Lang = Language, Visuo = Visuospatial, V.Mem = Verbal Memory, NV.Mem = Nonverbal Memory
