# Supplementary material for: Developing a Deep Brain Stimulation Neuromodulation Network for Parkinson Disease, Essential Tremor, and Dystonia: Report of a Quality Improvement Project
Source: PLoS One. 2016 Oct 6;11(10):e0164154. doi: 10.1371/journal.pone.0164154 (PMC5053513; doi:10.1371/journal.pone.0164154)
Supplement: S5 Appendix — (DOCX) [file pone.0164154.s005.docx]

**S5 Appendix**

MRI sequences used for patient selection and pre-operative stereotactic targeting. Post-operative DBS sequences are used for the localization of the lead tip.

|  | **Pre-operative DBS** | | | | |  | **Post-operative DBS*** | | |
| --- | --- | --- | --- | --- | --- | --- | --- | --- | --- |
| **PROTOCOL** | **SWI** | **DWI** | **AX T2 FLAIR** | **AX_3D_T1_**  **TFE** | **AX-COR_2D_ T2 TSE** | **AX_ 3D_ T1_ TFE** | | **AX_ 2D_ T2_ TSE** | **COR_2D_T2_**  **TSE** |
| **TR (ms)** | **16.2** | **5209** | **9000** | **8** | **2916.64** | **18** | | **2800** | **2800** |
| **TE (ms)** | **23.2** | **55** | **125** | **2.5** | **85** | **3** | | **122** | **122** |
| **FLIP ANGLE** | **90** | **90** | **90** | **< 15** | **< 90** | **28** | | **90** | **90** |
| **ECHO TRAIN** | **1 (3D-FFE)** | **80 (EPI)** | **30** | **238** | **21** | **15** | | **7** | **7** |
| **THICKNESS** | **1** | **4** | **3** | **1** | **2** | **1.5** | | **2** | **2** |
| **SLICE GAP** | **0** | **1** | **0.3** | **0** | **0** | **0** | | **0.2** | **0.2** |
| **FOV** | **23** | **23** | **23** | **26** | **26** | **26** | | **26** | **26** |
| **MATRIX** | **256 x 256** | **144 x 141** | **352 x 264** | **260 x 238** | **372 x 273** | **384 x 256** | | **384 X 256** | **384x256** |
| **PIXEL BANDWIDTH** | **172** | **1399/24** | **215** | **192** | **237** | **122** | | **50** | **50** |
| **SAR** | **NR** | **NR** | **NR** | **NR** | **NR** | **<0.1 W/Kg** | | **< 0.1 W/Kg** | **<0.1 W/kg** |
| **SCANNER** | **PHILIPS 3T** | **PHILIPS 3T** | **PHILIPS 3T** | **PHILIPS 3T** | **PHILIPS** | **Phillips 1.5T** | | **Phillips 1.5T** | **Philips 1.5T** |
| **SCAN TIME** | **3.50 mins** | **32 sec.** | **4.45 mins** | **9 mins** | **7-12 mins** | **13 mins** | | **12 mins** | **12 mins** |

*Post-operative DBS scans are performed on a 1.5T scanner with a Transmit/Receive head coil.

Ax—axial; COR—coronal; DWI—diffusion weighted images; EPI—echo planer; FFE—fast field echo; FLAIR—fluid attenuated inversion recovery; FOV—field of view; NR—no restrictions; SAR—Specific absorption rate; SWI—susceptibility weighted images; TE—echo time; TFE—turbo field echo; TR—repetition time; TSE—turbo spin echo
