# Supplementary material for: Developing a Deep Brain Stimulation Neuromodulation Network for Parkinson Disease, Essential Tremor, and Dystonia: Report of a Quality Improvement Project
Source: PLoS One. 2016 Oct 6;11(10):e0164154. doi: 10.1371/journal.pone.0164154 (PMC5053513; doi:10.1371/journal.pone.0164154)
Supplement: S6 Appendix — (DOCX) [file pone.0164154.s006.docx]

**S6 Appendix**

**Surgical Procedure**

On the day of the surgery, a Leksell G frame (Elekta Instruments, Atlanta, GA) is fixed with skull pins to the patient’s head after infusing the pressure points with 1% lidocaine. The frame is adjusted so that its base is approximately parallel to the plane of the anterior commissure – poterior commissure (AC-PC). Intravenous sedation (1-2 mg of midazolam) is given prior to advancing the skull pins symmetrically. A CT scan is obtained in the axial plane with a slice thickness of 1 mm and a field of view of 24 cm (General Electric Medical Systems, Milwaukee, WI). Preop MRI and CT digital images obtained the day of surgery (DICOM) are downloaded into a stereotactic surgical planning software package (Framelink, Medtronic, Inc), via a PACS network connection. Using mathematical paradigms, the MRI scan is merged with the stereotactic CT scan for targeting purposes.

***Stereotactic Localization of STN, GPi and VIM in fused MRI and CT images***. For all 3 targets we employ a combination of two procedures for locating the target sites: 1) indirect targeting which is based on identification of the AC-PC line, and 2) direct targeting based on direct visualization of the target or relevant adjacent structures.

For indirect targeting we use standard distances from the midcommisural point (MCP) to approximate the location of the desired target in all three planes. The distance between AC and PC can vary significantly between individuals and thus precise localization of these structures is essential for the accuracy of the target site. We use direct visualization of nuclear boundaries, mainly from T2 images, to make adjustments in the indirect coordinates to compensate for individual variations in nucleus locations.

The following coordinates are used for each of the target sites:

STN - 3 mm posterior to MCP, 12 mm lateral from the midline and 5 mm below the MCP. This oval shaped area can be seen in T2 TSE images passing through the anterior red nucleus (RN) in coronal sections, and immediately lateral to the RN in axial sections. We directly target the center of the hypointense structure.

GPi - 2-3 mm anterior to MCP, 21-22 mm lateral from midline and 4 mm below the MCP. These coordinates are checked on coronal T2 TSE images to ensure they are at the level of posterior mammillary bodies and directly above the optic tract.

VIM - 25% of the AC-PC length anterior to the PC, 55% of the AC-PC length lateral to midline or approximately 10mm from the wall of the third ventricle and at the level of MCP in the Z-plane. This nucleus is ill-defined on axial T1 MRI but in coronal T2 TSE images we aim for the midpoint between the lateral border of the medial dorsal thalamic nucleus and medial border of the internal capsule.

Once the target has been established, the trajectory is planned using the surgical planning software (Framelink). We aim for an anterior angle of approximately 60 degrees from the AC-PC in the sagittal projection and a lateral angle of 14-18 degrees in the coronal projection. The lateral angles are changed as needed to avoid passing through the lateral ventricles and through sulci to decrease the risk of deflecting the microelectrode and damaging cortical vessels respectively. The *x, y,* and *z* coordinates of the target site are obtained.

***Surgical procedure***. For PD patients, anti-parkinsonian medications are withheld at least 12 hours prior to the surgery to allow PD symptoms of tremor, rigidity, and bradykinesia to return, and to avoid medication induced dyskinesia during the surgical procedure. With the awake patient in a supine position on the OR table, the head frame is secured to the Mayfield headrest. The patient’s head is draped such that the face and body are accessible on the non-sterile side of the drape. Intravenous sedation is used as needed. Bilateral 14 mm burr holes are made anterior to the coronal suture, approximately 3.5 cm from the midline. The dura is coagulated, opened in a cruciate manner, and the underlying pial surface of the brain is coagulated with a bipolar cautery. The appropriate *x, y and z* coordinates are set on the stereotactic frame for the target site.

***Microelectrode Recording***. We use the Alpha-Omega Neuro-Nav (Nazareth, Israel) intraoperative microelectrode recording system for the neurophysiological localization of the target sites. A tungsten microelectrode (0.3-0.7 mΩ) is placed via a guide tube in the center of a ‘BenGun’ 5 microelectrode holder and lowered to a depth of 10 mm above the target with a microdrive connected to the Leksell frame. Microelectrode signals are band pass filtered (0.5 – 5 kHZ), amplified and displayed on the Neuro Nav system. Characteristic neurophysiological properties are used to identify each target site as follows:

*Subthalamic nucleus*: At a distance of 10 mm above target, neurons are encountered whose activity depends on the anterior-posterior and lateral approach angles. Typically our trajectory passes through the anterior thalamus, followed by a relatively quiet zona incerta (ZI) and fields of Forel, the STN, and finally the substantia nigra zona reticulata (ZR). However, if the STN approach is anterior and lateral the microelectrode will encounter no activity as it passes through the internal capsule. If the trajectory is more medial or posterior, the thalamic nuclei with low density and slow firing neurons will be encountered. Localization of the electrode within STN is evident when background noise dramatically increases indicating entrance into a region of high neuronal density. We have encountered 3 different populations of STN neurons: those that exhibit irregular firing patterns with rates of 30-50 Hz; those with regular bursting patterns in the 4-6 Hz range synchronous with resting tremor; and regular pacemaker populations with firing rates from 15-25 Hz. There is a somatotopic organization within the STN such that the leg territory is medial to the arm territory. Multiple studies suggest that the lateral anterodorsal aspect of the STN or dorsal ZI are the most clinically effective sites for therapeutic effects. The ventral border of the STN is marked by a dramatic decrease in background noise after which ZR neurons with a regular firing pattern and higher rates of discharge, ranging from 50 to 70 Hz, are encountered.

*Globus pallidus interna*: At a distance of 10 mm above target, neurons of the globus pallidus externa (GPe) are encountered. Two types of GPe neurons are identifiable: those with an irregular pausing pattern and firing rates of 50 Hz called ‘sputter’ cells and a smaller population with lower firing frequency rates of 18 ± 12 Hz. As the microelectrode leaves the GPe, the medial medullary lamina is crossed. The thickness of this neuron-free zone is used not only to indicate the passing from the GPe to the GPi, but also indicates whether the microelectrode is too lateral which occurs if the thickness of this fiber band is 4 to 6 mm. Passing into the GPi, neurons fire faster than GPe neurons at 80 ± 25 Hz with a more regular discharge pattern and shorter pauses. A second population consists of high-frequency bursting cells. As with the STN there appears to be a somatotopic organization within the sensorimotor postero-lateral regions of the GPi with the lower extremity represented more medially and dorsally than the upper extremities. This targeted area is bounded by the optic tract (OT) ventrally and the internal capsule medially. The optic track can be identified electrophysiologically by darkening the room and flashing a light into the patient’s eyes and listening for high frequency modulation of background audio signal coincident with light flashes. Additionally, microstimulation of the OT produces brief speckles or flashes of light of various colors. The internal capsule is identified by stimulation induced movement in the face or limbs. The presence of optic or corticospinal tracts responses is indicative of the need to move the microelectode position inferiorly and laterally respectively.

*Ventral intermediate nucleus of the thalamus (VIM)*: Electrophysiogically it is difficult to identify the VIM from the surrounding thalamic ventral oralis posterior (Vop) and ventral caudal nucleus (Vc). All three nuclei contain irregular high density cells and slow firing bursting neurons. However, we can accurately locate the VIM by identifying the Vc which demonstrates high amplitude evoked responses when the contralateral body is touched. Conversely, low threshold microstimulation resulting in paresthesia also helps to locate the Vc. Once the Vc is located, the microelectrode is moved 2-3 mm anterior into the VIM. The presence of bursting cells synchronous to tremor confirms the VIM location.

***Macrostimulation***. After the target site has been identified with MER, the tungsten microelectrode is replaced by a quadripolar platinium-iridium contact DBS lead (model 3387; Medtronic Inc, MN). Monopolar stimulation of each contact (with case positive) is tested in turn, with increasing voltages from 0 to 5 volts, with a fixed pulse width of 60 microseconds and frequency of 130 Hz. Adverse effects such as visual, motor, or cutaneous sensory responses to stimulation on structures that border the target are noted.

***Securing the lead***. When good clinical efficacy to macrostimulation is seen without adverse effects, the lead is secured to the burr hole using the Stimloc cap system (Medtronic Inc.). Fluoroscopic images are obtained before and after lead anchoring to assure that the lead has not migrated. Once the first lead is in place, MER and macrostimulation is repeated for the other side. Once both leads are locked in place, the distal ends of the leads are coiled up in a subgaleal pocket. All incisions are irrigated with antibiotic saline and closed. The patient is taken out of the stereotactic frame and a post-implantation CT scan is performed.

***Pulse generator placement***. One week after stage 1 of DBS surgery, the implantable pulse generator (IPG) is placed under general anesthesia. The patient is positioned supine with the head turned away from the side of the implant. Typically, the IPG is placed in a subcutaneous pocket below the clavicle, over the pectoralis fascia. The procedure consists of making 2 incisions: one below the clavicle to hold the IPG and another posterior to the ipsilateral ear for placement of the connectors between the distal ends of the DBS leads and the extension cables which connect the DBS leads to the IPG. For a single PC IPG, two 40 cm extension cables are tunneled from the subclavicular pocket to the retroauricular incision. The DBS leads are connected to the extension cable using a standard four-screw connector, which is protected by a silastic sheath and placed in the subgaleal pocket. The distal ends of the extension cables are then connected to each SC IPG. In the case of 2 SC IPGs (one below each clavicle), one 40 cm and one 60 cm extension lead is used with the longer extension lead connecting the contralateral DBS lead.
